# VIP in construction: systematic development and evaluation of a multifaceted health programme aiming to improve physical activity levels and dietary patterns among construction workers

**DOI:** 10.1186/1471-2458-12-89

**Published:** 2012-01-30

**Authors:** Laura Viester, Evert ALM Verhagen, Karin I Proper, Johanna M van Dongen, Paulien M Bongers, Allard J van der Beek

**Affiliations:** 1 Department of Public and Occupational Health and the EMGO+ Institute for Health and Care Research, VU University Medical Center, Amsterdam, The Netherlands; 2 Body@Work, Research Center Physical Activity, Work and Health, TNO-VU University Medical Center, Amsterdam, The Netherlands; 3 Department of Health Sciences and the EMGO+ Institute for Health and Care Research, Faculty of Earth and Life Sciences, VU University Amsterdam, Amsterdam, The Netherlands; 4 Netherlands Organisation for Applied Scientific Research, TNO, Hoofddorp, The Netherlands; 5 Department of Public and Occupational Health and the EMGO+ Institute for Health and Care Research, VU University Medical Center, Van der Boechorststraat 7, 1081, BT Amsterdam, The Netherlands

**Keywords:** Obesity/overweight, Musculoskeletal disorders, RCT, Energy balance related behaviour, Physical activity, Dietary behaviour, Construction workers, Intervention mapping

## Abstract

**Background:**

The prevalence of both overweight and musculoskeletal disorders (MSD) in the construction industry is high. Many interventions in the occupational setting aim at the prevention and reduction of these health problems, but it is still unclear how these programmes should be designed. To determine the effectiveness of interventions on these health outcomes randomised controlled trials (RCTs) are needed. The aim of this study is to systematically develop a tailored intervention for prevention and reduction of overweight and MSD among construction workers and to describe the evaluation study regarding its (cost-)effectiveness.

**Methods/Design:**

The Intervention Mapping (IM) protocol was applied to develop and implement a tailored programme aimed at the prevention and reduction of overweight and MSD. The (cost-) effectiveness of the intervention programme will be evaluated using an RCT. Furthermore, a process evaluation will be conducted. The research population will consist of blue collar workers of a large construction company in the Netherlands.

**Intervention**

The intervention programme will be aimed at improving (vigorous) physical activity levels and healthy dietary behaviour and will consist of tailored information, face-to-face and telephone counselling, training instruction (a fitness "card" to be used for exercises), and materials designed for the intervention (overview of the company health promoting facilities, waist circumference measuring tape, pedometer, BMI card, calorie guide, recipes, and knowledge test).

**Main study parameters/endpoints**

The intervention effect on body weight and waist circumference (primary outcome measures), as well as on lifestyle behaviour, MSD, fitness, CVD risk indicators, and work-related outcomes (i.e. productivity, sick leave) (secondary outcome measures) will be assessed.

**Discussion:**

The development of the VIP in construction intervention led to a health programme tailored to the needs of construction workers. This programme, if proven effective, can be directly implemented.

**Trial registration:**

Netherlands Trial Register (NTR): NTR2095

## Background

The worldwide prevalence of overweight and obesity is increasing at a high rate. This also affects the Dutch population, where in 2009, according to the Central Bureau of Statistics Netherlands (CBS), more than 50% of the male population and 40% of the female population was overweight [body mass index (BMI) ≥ 25 kg m^-2^] [1]. Of this population 11% of the men and 12% of the women were obese (BMI ≥ 30 kg m^-2^). Excess body weight is associated with increased mortality and morbidity rates. To illustrate, obesity has a short-term negative impact on health, e.g. musculoskeletal disorders [2-5], as well as long-term consequences, e.g. diabetes mellitus type II and cardiovascular disease [6,7]. In addition to health-related problems in the individual, overweight and obesity are related to work-related measures, such as increased sick leave and decrease of productivity [8-14]. More than 10% of sick leave and productivity loss at work may be attributed to lifestyle behaviours and obesity [14]. Consequently, the economic consequences of overweight and obesity are high. In the Netherlands the annual direct costs have been estimated at €500 million, approximately 2% of the total national health care costs [15]. However, the indirect costs resulting from work absence and work disability related to overweight and obesity are estimated at €2 billion [16].

Recent data obtained from periodic health screenings among 39,400 construction workers showed that the prevalence of overweight and obesity in construction workers is higher than in the general Dutch adult population. Of all construction workers 63% is overweight and 15% is obese [17]. It is argued that within this specific population negative health-related lifestyle factors (e.g. low levels of daily life physical activity, smoking, and dietary patterns) are more prominently present than in the general population. Furthermore, the average age of construction workers has been steadily increasing in the past decade, and will do so in the decade ahead. As a result, employee health is an important concern for the construction industry, both from a corporate social responsibility as well as a risk management view. Fit and healthy employees working in a healthy environment are of critical importance to realise organisational goals. Operating in a highly competitive business environment with increasing pressure on the labour market, and an aging workforce, employers are becoming aware that they need to implement measures to improve productivity and efficiency, and to invest in the health of their employees.

Workplace health promotion has been shown to play a major role in achieving such outcomes; directly by educating the workforce and providing opportunities for physical activity, and indirectly by influencing social norms [18]. Workplace health promotion may constitute of a diverse set of health promoting activities, such as periodic health screenings (PHS), courses in smoking cessation, and enhanced access to physical activity. Many employers are offering such fringe benefits to their employees. However, the health enhancing effects of these facilities are not yet identifiable and it remains unclear whether the actual group of workers at risk is being reached. It has been argued that these facilities are predominantly used by the healthy part of the workforce. Therefore, in order to increase effectiveness it is crucial to provide a supporting health promotion programme that promotes the utilisation of the offered health enhancing facilities by employees with lifestyle-related risk factors for disease. The overall aim of this study is to develop and evaluate such a supporting health promotion programme (VIP in Construction). More specifically the current study aims to systematically develop a tailored intervention programme for the prevention and reduction of overweight and musculoskeletal disorders (MSD) in construction workers and to describe the evaluation study regarding the (cost-)effectiveness of this programme.

## Methods

The present study consists of 2 phases. In the first phase a health enhancing intervention was developed, tailored specifically to the possibilities, needs and wishes of the management and employees of the participating construction company. The second phase of this study involves the evaluation of the intervention.

**Figure 1:**
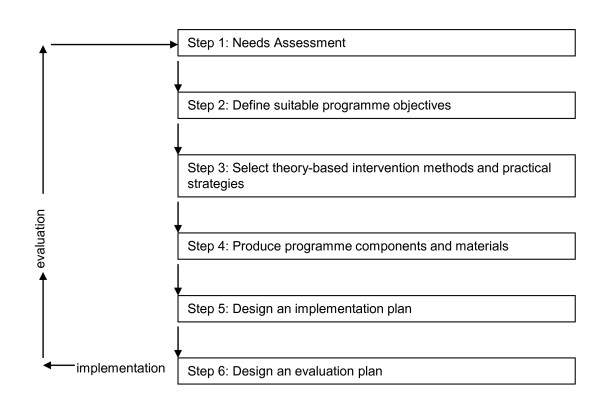
**Steps of the Intervention Mapping process**.

The "VIP in construction" intervention was systematically designed based on the Intervention Mapping (IM) protocol [19]. IM describes a process for developing theory- and evidence-based health promotion programmes, and involves a systematic process that prescribes a series of six steps: (i) performing a needs assessment; (ii) defining suitable programme objectives; (iii) selecting theory-based intervention methods and practical strategies; (iv) producing programme components and materials; (v) designing an implementation plan; and (vi) designing an evaluation plan (Figure 1). Collaboration between the developers, the users of the intervention and the target population is a basic assumption in the IM process [19]. This paper describes in detail the development of a health enhancing intervention programme for construction workers by using the steps of the IM process. Step 6 of the process describes in detail how the (cost-) effectiveness of the developed programme will be evaluated.

### Phase 1: Intervention development

#### Step 1: Needs assessment

Literature was reviewed and interviews, questionnaires, and focus group interviews with management, employees and other stakeholders were carried out. This provided insight into the ruling health issues, underlying risk factors (behaviour and environmental conditions), and determinants of the underlying behaviours. In addition, the reach, success and failure factors of current company health promotion activities were summarised. This needs assessment results in the formulation of programme outcomes.

### Health problem and target group

The target group for this intervention was specified as all blue collar workers of a construction company. From interviews with the management of the company and from information obtained from Occupational Health Services (OHS) reports it was concluded that the main health concerns for the target population are overweight and MSD. In general, in the construction industry MSD are the primary reason for long-term sickness absence and disability [20,21]. Also the company records show that long-term sickness absence among blue collar workers is mainly caused by MSD.

Especially in professions with heavy physical demands, such as those in the construction industry, muscle fatigue or musculoskeletal discomfort may be perceived during work and may eventually result in musculoskeletal pain [22]. Several work-related physical factors have been identified that can increase the risk of musculoskeletal pain among workers [22-27]. Besides work-related factors, health-related factors, such as obesity may play a role in musculoskeletal pain. Findings of a meta-analysis on the association between obesity and low back pain indicate that overweight and obesity increase the risk of low back pain [5]. In a cohort study of construction workers [28] it was found that MSD represent the most frequent cause of work disability and that obesity increased this risk. Since overweight and MSD are possibly associated, the intervention will aim at addressing these health problems together.

### Key determinants & risk factors for overweight and MSD

Literature was reviewed to identify which theoretical constructs best predict overweight and MSD.

Energy-balance-related behaviour is an important factor to consider in the development of health interventions aiming at healthy lifestyle. Weight gain, overweight, and obesity have been associated with various specific behaviours related to diet and physical activity. Risk factors for obesity are considered to be: sedentary lifestyles (i.e., time spent sitting), a high intake of energy-dense high-fat and low-fiber diet, consumption of sugar-sweetened soft drinks, frequent snacking, and large portion sizes [29,30]. Protective factors against obesity are considered to be: regular physical activity and consumption of a high-fiber diet (for instance, a diet high in fruits and vegetables) [29,30].

MSD have a multifactor origin, several work-related and non work-related risk factors contribute to their development [22,31,32]. According to the model of workload and capacity by Van Dijk et al. [33], health effects may result from an imbalance between workload and capacity. A prospective study of Hamberg-van Reenen et al. (2006) [34] confirmed that an imbalance between physical capacity and exposure to work-related physical factors was a risk factor for future musculoskeletal pain. For example, it is generally assumed that for workers with high muscle strength, high exposure to physical factors may result in less musculoskeletal pain than for workers with low muscle strength [35].

### Questionnaire and focus group interviews

In order to be relevant, the intervention needs to account for the lifestyle habits and preferences of the target group. Therefore, to obtain information on specific dietary and physical activity behaviour in the target group, a short questionnaire was completed by a sample of 42 construction workers. These specific behaviours were further discussed in the focus group interviews. The aims of the focus groups were: identifying the main and modifiable determinants of the lifestyle behaviours (physical activity and diet), risk factors for MSD, and the reach and participation of the current company health promoting activities. Also, input from the focus group interviews was used to determine the content and design of the intervention. A total of 8 focus group interviews with construction workers (n = 62) were carried out. The focus group interviews were held at different worksites of the company to reach workers from different professions, and participants were randomly selected to avoid getting input only from workers who are already motivated to participate in health programmes.

#### Risk factors and determinants for the health problems

Health beliefs and health behaviours related to diet and physical activity were discussed in focus group interviews. From the focus group interviews it could be concluded that workers have some basic knowledge of nutritional standards, but they are not aware of their personal intake levels. The methods most often listed by the construction workers to improve their energy balance were less snacking and reducing alcohol consumption. Further solutions mentioned: decreasing intake of sugar-sweetened beverages or replacing them with healthier options, increasing fruit intake, and decreasing dinner portion size. From the focus group interviews we also learned that, in general, the workers' partner mainly determines the food choice at home, and the workers preferred to get personalised information on diet, as opposed to general information.

The interviewed workers indicated that they believed that their work activities provided enough physical activity. However, from periodic health screening data [17] it is clear that a substantial percentage of workers still do not reach healthy levels of physical activity according to the Nederlandse Norm Gezond Bewegen (NNGB) (33%) and the guideline to achieve a good fitness level (Fitnorm) (80%). According to physical activity guidelines these levels should be achieved to improve and maintain health [36].

Workplace physical demands, such as manual material handling (lifting heavy objects), extreme weather and workplace conditions (uneven terrain, awkward working postures), work pace and planning were most mentioned to be risk factors at work for developing MSD. Also behavioural risk factors were mentioned, such as not taking enough rest-breaks during work, wrong work posture, and wrong use of (ergonomic) work aids. A social/managerial factor that was considered important was poor communication between supervisors and the workers concerning problems or solutions for prevention or reduction of MSD in combination with perceived barriers for addressing those problems.

#### Intervention input from focus group interviews

Although poor physical fitness was not frequently mentioned as one of the risk factors for MSD in the focus groups, improving physical capacity was mentioned as a possible preventive measure or solution. According to the literature increasing vigorous physical activity (PA) is a preventive method that targets body weight control as well as MSD [37-42]. Strong evidence was found for the effectiveness of workplace physical activity programmes in increasing strenuous physical activity levels as well as in preventing MSD [43].

To design a feasible intervention programme, the reach of current company health promoting activities and the requirements and design for an intervention programme were also discussed in focus group interviews. From the interviews amongst employees it could be concluded that the current health promoting activities were not optimally reaching the workers. The most important reason indicated by the interviewees was that workers were not aware of the present prevention practices, i.e. that these were not communicated in the right way. Also those who were aware of the possibilities (e.g., the reduction of gym membership fees) were often under the impression that these measures were mainly initiated for office workers of the company. From the interviews it became clear that communicating the health promoting activities in a suitable manner for the target group should be an important objective for the intervention programme.

Furthermore, workers were asked about the necessary requirements and design for an intervention programme in order to reach non-participants and motivate them to participate in prevention programmes. Workers argued that an intervention programme should focus on communicating personal health risks, since perceived health was considered to be a necessary motivator for changing behaviour. From the focus group interviews we learned that the regular company periodic health screening (PHS) was generally seen as a positive starting point for discussing lifestyle. However, during the PHS there is often not enough time to discuss the outcomes. It became clear that linking the intervention to the PHS could improve participation to worksite health promoting activities.

### Programme objectives and outcomes

The needs assessment indicated that the intervention should address both dietary habits and physical activity with the overall programme objective being the prevention and reduction of overweight and MSD among construction workers. In addition, to specifically target and prevent MSD by improving physical capacity, workers could be stimulated to increase their general physical activity by means of specific exercises, sports, and daily physical activities during leisure time.

Based on literature and focus group input, intervention strategies to prevent or reduce MSD could focus on (1) increasing physical capacity by improving general physical activity or specific exercises and/or (2) decreasing workload. However, there was no management support for implementing strategies aimed at decreasing workload. The management indicated that other company projects have already started considering physical workload; therefore decreasing workload is not a programme objective for the VIP in construction intervention.

The risk behaviours described in the needs assessment were translated into health-promoting behaviours. The health behaviours that should be targeted were then formulated in programme outcomes of the VIP in construction intervention, and are presented in Table 1.

**Table 1:** Programme outcomes

	Programme outcomes
1)	Energy intake quantity: Workers reduce their energy intake by decreasing portion size and alcohol consumption

2)	Energy intake quality: Workers replace energy dense products by healthier options (fibre rich products and beverages without sugar)

3)	Energy output quantity: Workers increase their levels of physical activity

4)	Energy output quality: Workers perform specific exercises to prevent or reduce MSD

#### Step 2: Performance objectives, determinants, and change objectives

Step 2 provides the foundation for the intervention programme by specifying who and what will change as a result of the intervention. The product of this step is a set of matrices that combines performance objectives with selected personal and external determinants to produce the target of the intervention (change objectives).

### Performance objectives

The programme outcomes formulated in the needs assessment were translated into performance objectives: what do the participants have to do to accomplish these outcomes? Based on the self-regulation theory and determinants for behaviour obtained from literature and focus group interviews, performance objectives were stated for each of the programme objectives. As an example, the performance objectives for the third programme objective are illustrated in Table 2.

**Table 2:** Performance objectives

**Performance objective related to Programme Outcome 3: "Workers increase their levels of physical activity**"
Workers should:
1) Self-monitor physical activity
2) Set goals to increase physical activity levels
3) Form implementation intentions
4) Implement healthy levels of physical activity
5) Evaluate personal goals

### Determinants of behaviour change

**Figure 2:**
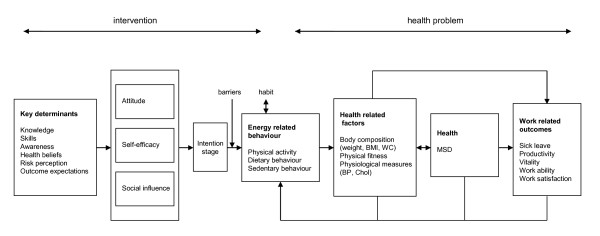
**Conceptual model of the VIP in Construction intervention**.

IM states that for health promotion intervention development, instead of searching for predictors of present behaviour, health-related behaviour (e.g. high energy intake) should be translated into a health-promoting behaviour or behaviour change (e.g. energy intake reduction) and then search for determinants of the required change. The determinants for the performance objectives in this study were based on literature review and focus group interviews and were selected on importance and changeability for the specific target group. The following personal and external determinants for physical activity were identified: skills, self-efficacy, attitudes, barriers, habits, outcome expectations, resources, awareness, risk perception, and health beliefs. For dietary behaviour, the following personal and external determinants were selected for this intervention: knowledge, awareness, risk-perception, health beliefs, habits, and social support. The conceptual model of the VIP in construction intervention is described in Figure 2.

### Change objectives

Change objectives were created by crossing performance objectives with determinants in a matrix. An example of the matrix for performance objective 3 is given in Table 3.

**Table 3:** Selected change objectives for performance objective 3

Performance Objectives	Skills and self-efficacy	Awareness and attitudes	Outcome expectations
PO.3. "Workers increase their levels of physical activity (by increasing PA of vigorous intensity and decreasing sitting time)**"**		A.3 Express positive attitude towards increasing levels of physical activity	OE.3. Expect that increasing levels of physical activity will have positive health outcomes

PO.3.1 Self-monitor physical activity	SSE.3.1 Know how to self-monitor PA	A.3.1 Express positive attitude towards self monitoring of PA	

PO 3.2. Set goals to increase physical activity levels	SSE.3.2 Express confidence for setting goals to increase PA levels	A.3.2 Express positive attitudes towards goal setting	OE.3.2. Expect that goal setting will increase PA levels

#### Step 3: Methods and strategies

After constructing the change matrices, the next step was to select appropriate theoretical methods for behaviour change and to translate these into practical strategies.

### Theory-based intervention methods

For each determinant (e.g. self-efficacy, skills, knowledge, social support) appropriate theoretical methods were identified from literature and from guidance of Bartholomew et al. (2006) [19]. Theoretical input for these methods and strategies was derived from behavioural theory literature. This includes health behaviour models (theory of planned behaviour (TPB) [44] and the health belief model (HBM) [45]) as well as behaviour change models (transtheoretical model (TTM) [46] and the precaution adoption process model (PAPM)[47]). Decisions about suitable strategies were made based on feedback of key contacts within the organisation, and focus group data. These were then translated into strategies suitable for implementation in the workplace. The results of this step are presented in Tables 4 and 5.

**Table 4:** Methods and strategies selected for dietary behaviour (programme outcomes 1&2)

Determinant	Theoretical Methods	Strategy	Tools/Materials
*a) Personal *			

**Knowledge**	Passive learning/providing information	Providing written and/or verbal information	Tailored brochures

	Active processing of information		Knowledge tests

**Awareness of personal intake levels**	Self-evaluation	Comparing intake in relation to standards	Worksheet self-test on healthy standards

	Feedback	Feedback on intake levels	Personal feedback PHC

**Habits**	Implementation intentions (goal setting)	Formulation of specific personal intentions	PHC assists in formulating practical goals + PEP form

**Awareness, risk perception & health believes**	Information about personal risk	Personalized risk feedback from health screening	Expert monitoring and evaluation of BMI, waist circumference, blood pressure, behaviour etc. in relation to healthy standards (PHC)
	Scenario-based risk information	Providing tailored risk information on long-term effects and information on benefits of healthy behaviour	Tailored brochures
	Re-evaluation, self-evaluation, and consciousness raising	Awareness of own body composition by self-monitoring	Waist circumference measuring tape BMI card
		Delivering information on the relationship between calories & PA	Calorie guide (# min PA required to lose a certain amount of calories)

*b) External*			

**Social support**	Mobilising social support from spouse/family	Providing healthy recipes tailored to target population	Test recipes

**Table 5:** Methods and strategies selected for PA (programme outcome 3&4)

Determinant	Theoretical Methods	Strategy	Tools/Materials
*a) Personal*			

**Self- Efficacy**	Goal setting	Formulation of implementation intentions	Worksheet (PEP form) + PHC assists in goal setting
	Reinforcement	Evaluation of change process	Follow-up contacts PHC

**Attitudes**	Feedback	Provide personal feedback	PHC provides feedback on (perceived) positive consequences of PA

**Skills**	Guided practice	Instruction/skills training	Training instruction exercise card (core stability & strength)

**Habits**	Implementation intentions (goal setting)	Formulation of specific personal intentions	Worksheet (PEP form) + PHC assists in goal setting

**Awareness, risk perception & health believes**	Information about personal risk	Personalized risk feedback from health screening	Expert monitoring and evaluation of BMI, waist circumference, blood pressure etc. in relation to healthy standards
	Scenario-based risk information	Providing risk information on long-term effects and information on benefits of healthy behaviour	Tailored brochures
	Re-evaluation, self-evaluation, and consciousness raising	Awareness of own energy balance (PA) behaviour	Pedometer
		Delivering information on the relationship between calories & PA	Calorie guide (energy balance information # min PA required to lose calories)

*b) External*			

**Perceived physical environment**	Promotion/facilitation	Providing information on workplace health promotion	PHC provides (contact) information on the companies facilities and cost reduction

### Practical strategies

Literature was reviewed to identify which strategies are most frequently found as part of successful interventions aimed at increasing (vigorous) physical activity and improving dietary habits. Synergies between diet and exercise in modifying body composition have been reported [48,49]. Furthermore, a combination of interventions on physical activity and dietary habits were found to be more (cost-)effective than interventions on physical activity alone [50].

A review on determinants of participation in worksite health promotion programmes showed that programmes that offer a multi-component strategy and focus on multiple behaviours have a higher overall participation level [51]. When targeting multiple lifestyle behaviours, identifying an individual's stage-of-change on behaviour can help to determine which behaviours an individual should be targeted for change (at various points) in the intervention [52]. The stage-of-change construct can facilitate tailoring of interventions by matching intervention strategies to individuals' motivational readiness. Furthermore, in weight management in which multiple diet and activity changes can achieve weight change, individuals may be more motivated to change some specific behaviours than in others. Therefore, participants should be able to choose which behaviour they intend to change.

A strategy for increasing risk awareness could be feedback on health screening. The review of Soler et al. 2010 [53] indicates that assessment of health risks with feedback is useful as a gateway intervention to a broader worksite health promotion programme that may include a set of health promotion activities to improve the health of employees. The workers indicated in the focus group interviews that there often is no sufficient follow-up or feedback during or after the PHS. Standardised follow-up is available only in the case of high risk (for example high blood pressure). Also, as a preventive measure, feedback and personal information could be very important to induce behaviour change [54,55]. This was also found to be effective in construction workers [56]. Therefore, personal counselling with extra feedback for behaviour change should be an important element of the intervention.

#### Step 4: Producing programme components and materials

In this step of the IM process methods and practical strategies are translated into programme components and materials. The starting point of the intervention should be informing the employees about the company health promotion activities. Personal health coaching and information materials should be added to the current health promoting activities of the company to include all determinants of the formulated programme objectives.

### Programme description

The intervention will take place during a 6-month period and will consist of materials and tailored information on physical activity and diet, personal health coaching (PHC), and training instruction. Both the PHC protocol and specific materials were developed to be able to connect the intervention to the PHS and tailor the intervention to the needs (individual risk factors) and wishes of the participants. Based on the baseline measurements and questionnaires a quick scan will be applied to tailor the intervention to the participants. Tailoring variables will be health indicators (BMI and waist circumference), current lifestyle behaviour (physical activity) and stage-of-change (for physical activity as well as dietary behaviour).

### Programme materials

The programme materials were made attractive and recognisable for the target group by using a standard lay-out and logo. The "VIP in Construction toolbox" will consist of tailored brochures, a calorie guide, a pedometer, a BMI card and waist circumference measuring tape, recipes and a knowledge tests, an overview of the company health promoting facilities, PEP forms, and an exercise card. The exercises will consist of strengthening and stabilization exercises for the abdominal and dorsal muscles and will be well described on an exercise card. The exercises should be performed 3 times a week. The participants will receive instruction for the use of the exercise card from the PHC. The exercises on the card should be easily fitted in daily life routines; participants should be able to perform the exercises at home, and without any use or purchase of materials which potentially enhances compliance.

### PHC

The coaching contacts will specifically aim at the programme outcomes as formulated in the needs assessment. The coaching contacts will consist of the following elements: 1) feedback, 2) goal setting, 3) feedback on formulated goals, 4) instructions for self-monitoring, and 5) training instruction.

1) The participants will receive additional feedback on their health screening and current lifestyle behaviour.

2) The PHC will support in goal setting, by helping the participants in formulating a personal motivation and action plan. These plans will contain physical activities, healthy food choices or a combination. Participants will be encouraged to target behaviour that is not at the desired level. Questions will be asked on what participants want to change, and they will be asked to formulate and write down specific goals and strategies to change the behaviour. In addition, information about the company's health promoting activities will be given and the intervention materials will be distributed and clarified.

3) Feedback on formulated goals will be given during the follow-up contacts. The PHC will keep a record of the goals and plans of the participant; in the follow-up contacts these goals should be evaluated. Possible barriers should be discussed and/or new goals should be formulated.

4) Participants will receive instructions for self-monitoring by using the PEP forms and materials.

5) The PHC will give instructions how to use the exercise card.

During the intervention, participants will be coached face-to-face in formulating their personal motivation and action plan. Follow-up contacts (feedback and motivating) will be conducted by telephone. The number and duration of contacts will vary with the outcome of the quick scan, with a minimum of 2 and a maximum of 4 contacts. The number of contacts (A, B, C) will be determined by a participant's stage-of-change (for physical activity as well as dietary behaviour). An overview of the contacts is given in Table 6. A web-based system will be used to register the participants' appointments, follow-up contacts, and content of the contacts (goals & action plans).

**Table 6:** Coaching contact schedule

PHC contacts	2 weeks after baseline measurements	1 month	2 months	3 months	4 months
A	Intake (60 min face-to-face)	Follow-up 1: (30 min; telephone)	Follow-up 2: (15 min; telephone)		Follow-up 3: (15 min; telephone)

B	Intake (60 min face-to-face)		Follow-up 1: (30 min; telephone)	Follow-up 2: (15 min; telephone)	

C	Intake (30 min face-to-face)			Follow-up 1: (10 min telephone)	

#### Step 5: Adoption & implementation plan

The product of step 5 is a plan for accomplishing programme adoption and implementation by influencing behaviour of individuals who will make decisions about adopting and using the programme and the individuals who deliver the programme.

#### Company involvement

To gain insight into facilitating factors and possible barriers regarding the adoption and implementation, management and (potential) users of the programme were interviewed. The human recourse management was involved in the programme development from the start to ensure top-down adoption in the organisation and increase of the chance of long-term implementation. During the intervention period the process will be monitored for unforeseen difficulties and possible barriers in adoption. Also a communication plan was written for the company. The main goal of this communication plan was to inform the target group and the management about the project and to obtain support from the direct management.

#### Participants' compliance (important factors to encourage the adoption of the intervention by the participants)

To decrease barriers for participation, communication to the participants will be performed in cooperation with their employers, to show company involvement and support for the programme. Furthermore, the invitation to the study will be done simultaneously with the invitation to the PHS, to adapt the programme to the regular procedures. To make participation feasible for the participants the follow-up measurements as well as the first face-to-face contact with the coach will take place at the worksite and during work hours.

In the planning of the programme, the planning of regular health screening was taken into consideration. Based on de schedules of the health screening, it was decided that the recruitment for the intervention should last at least 12 months, to ensure exposure to all the companies' business units, and worker age groups.

The participating occupational physicians (OP) and nurses received instructions during a kick-off meeting as well as by e-mail and telephone, as they will have an important role in linking the intervention to the PHS and motivating the workers to participate. To ensure that a standardized protocol will be used by the PHCs, all coaches received a manual describing the protocol and goals for the coaching sessions in detail. Just before the start of the intervention a training session will be held.

### Phase II evaluation

#### Step 6: Evaluation plan

##### Study design

The effectiveness of the programme will be measured by performing an RCT. Participants will be measured at baseline (T0), at 6 months (T1), and at 12 months (T2). Consenting participants will be randomised to the intervention or control group after the baseline measurement. The control group will receive care as usual and will only be contacted for the baseline and follow-up measurements. The study design and procedures have been approved by the Medical Ethics Committee of the VU University Medical Centre.

##### Study population and setting

The research population will consist of all blue collar workers of a construction company. This will include construction site workers as well as factory workers of the company. The recruitment of participants will be conducted through the usual communication channels of the company at a non-compulsory PHS.

##### Power calculation

Sample size was based on detecting a difference in change in body weight between the intervention and the control group. In each group (intervention and control) 130 participants will be needed, based on a power of 80% and an alpha of 5%, and an expected weight loss of 1.5 kg (sd 4.3 kg) as result of the intervention. The used standard deviation was subtracted from previous work from our research group, studying construction workers [56]. Taking into account a loss to follow-up of 20%, 324 workers should be included in this study.

##### Randomisation

Randomisation will take place at an individual level. After baseline measurements the participant will be randomly assigned to either the intervention or the control group by a computer generated list using SPSS (version 15). The randomisation will be prepared and performed by an independent researcher (i.e. the research assistant).

##### Measurements

Assessment of the study parameters will be done using a combination of questionnaires and physiological measurements. Part of the study parameters will be obtained from physical examinations and questions on outcome measures are based on questions used for the PHS survey in the construction industry. In the Netherlands, this survey is widely used and tested on validity among construction workers who participate in PHS.

Together with the invitation for this company PHS, all workers will receive a brochure about the study, an informed consent form, and an additional questionnaire in order to measure those variables not included in the PHS. For each study parameter, the following paragraphs describe how it will be measured for this study.

### Primary outcome measures

#### Body composition

##### Body weight and BMI

Body weight and height will be measured at the OHS by the occupational physician or the assistant during the PHS. Weight will be measured using a digital weight scale. Body weight and height will be measured with the participants standing without shoes and heavy outer garments. Data on body weight and height will be used to calculate Body Mass Index (BMI) (kg/m^2^).

##### Waist circumference

BMI does not give insight into body fat distribution; therefore waist circumference will be measured as an indicator of health risks associated with visceral obesity [57]. Waist circumference will be measured during the PHS by the OP or assistant as midway between the lower rib margin and the iliac crest with participants in standing position at the end of expiration [58]. To standardize waist circumference measurement, OPs and assistants will be provided with a Seca 201 waist circumference measure (Seca, Hamburg, Germany) and measuring protocol.

### Secondary outcome measures

#### Musculoskeletal disorders (MSD)

The prevalence of MSD will be assessed using questions derived from the PHS. Using a dichotomous scale (yes/no), questions relate to the prevalence of regular pain or stiffness in both the upper and lower extremity regions. Additionally, using the validated Dutch Musculoskeletal Questionnaire [59], the prevalence of MSD during the past three months will be measured for the different body regions. The intensity of pain will be measured using Von Korff scales [60]. Workers will be asked to indicate their intensity of pain (i.e. average pain and worst pain experienced) on an 11-point numerical scale (0-10).

#### Energy balance-related behaviour

##### Physical activity

The frequency of vigorous activities will be obtained from the PHS questionnaire and moderate physical activity will be assessed by the number of days per week moderate intensity activities are performed (such as walking and cycling) for at least 30 minutes. These questions relate to international physical activity guidelines [61] as well as to the Dutch guidelines [62]. Additionally, the validated Short Questionnaire to Assess Health enhancing physical activity (SQUASH) will be applied [63]. The SQUASH measures duration, frequency and intensity of different domains of physical activity (active work transportation, occupational physical activity, household activities, and leisure time activities). Data from the SQUASH will be expressed as energy expenditure in METminutes per week.

As a complementary method, physical activity and sedentary behaviour will be assessed objectively using accelerometers in a random sample of 50 participants of both the intervention (n = 25) and control group (n = 25). This random sample will wear an accelerometer (Actigraph) during 7 consecutive days. The accelerometer will register the actual physical activity during and outside work hours.

##### Dietary intake

Alcohol consumption will be obtained from the PHS questionnaire asking participants to report their average consumption (in glasses per week). Portion size at dinner, number of beverages and slices bread, as well as consumption of energy dense snacks will be assessed using questions that were also used in the Health under Construction study [64]. Average weekly intake and daily portions of several food groups during a usual week during the past month are indicated in these questions. Fruit and vegetable consumption will be measured using the validated Short Fruit and Vegetable questionnaire (validity r = 0.50) [65]. The number of days per week and the number of daily servings of fruit, vegetables and fruit juice will be measured using five items on citrus fruit, other fruits, cooked vegetables, raw vegetables, and fruit juice.

#### Determinants of energy balance-related behaviour

The intervention will aim at improving energy balance-related behaviour (physical activity and dietary behaviour). Personal coaching and feedback will be tailored to self-efficacy and stage-of-change. Therefore, it is necessary to measure these constructs for physical activity and dietary behaviour. Based on models of behaviour and behaviour change, questions will be asked on knowledge, attitudes, self-efficacy and stage-of-change for physical activity and dietary behaviours [46,47].

#### Health-related measures

##### Self-reported Physical Functioning

Subjective physical functioning will be measured using the RAND-36 [66,67]. The RAND-36 health survey is a widely known and reasonably reliable and valid measurement of health-related quality-of-life [68]. The RAND-36 consists of 36 questions, with clusters of: physical functioning, social functioning, role limitations (physical problem), role limitations (emotional problem), mental health, pain, general health perception, and health change. In the present study, the validated Dutch version will be used.

##### Fitness

Although maximal volume of oxygen consumption (VO_2_max) is considered the gold-standard for measuring aerobic capacity, its measurement requires strict protocols and trained personnel. For this study fitness will be measured by using a non-exercise test estimation model including age, BMI, resting heart rate, and self-reported physical activity [69,70].

##### Cardiovascular disease (CVD) risk profile

CVD risk profile will be assessed using the European Systematic Coronary Risk Evaluation (SCORE) [71]. The SCORE is based on the CVD risk variables smoking, systolic blood pressure, and blood cholesterol levels (either total cholesterol or the ratio total/HDL cholesterol). All variables will be measured by the OP or the assistant during the PHS. Blood cholesterol (mmol/l) will be measured by taking a venous blood sample. The SCORE will be filled in based on blood pressure and cholesterol levels, as assessed in the medical examination and smoking behaviour as assessed in the PHS questionnaire.

#### Work-related measures

##### Workplace productivity loss

Sickness absence data (work absenteeism) will be collected from company records. Presenteeism (reduced productivity while at work) will be measured using the WHO Work Performance Questionnaire (WHO-HPQ) [72,73] and the PROductivity and DISease Questionnaire (PRODISQ) [74]. Participants will be asked to complete these questionnaires at 3, 6, 9, and 12 months.

##### Work ability

For companies work ability is an indicator of the productivity of its own human resources. Work ability will be assessed by the Work Ability Index as measured in the PHS questionnaire.

##### Work engagement, work satisfaction & vitality

Vitality will be assessed by the six items of the Utrecht Engagement Scale (UWES) that refer to high levels of energy and resilience, the willingness to invest effort, not being easily fatigued, and persistence in the face of difficulties [75]. In addition, work related measures such as organisational commitment and work satisfaction will be evaluated.

##### Use of company facilities

Since the intervention aims to increase the use of company health promoting facilities (e.g. company sponsored fitness), the use of these facilities will be reported by the participants at 6 and 12 months.

#### Cost measures

##### Intervention costs

These include the costs for the "VIP in Construction toolbox" and the PHC. PHC costs include costs for the health coach, housing costs, costs for printed materials, and travel expenses of the PHC. Since the PHC contacts will take place during work hours, the costs of lost productivity due to the intervention will be included as well. Coaches will record the frequency and duration of the face-to-face and telephone contacts. Intervention costs will be valued using a bottom-up approach.

##### Other workplace health promotion costs

The use of company facilities will be valued using invoices of contractors.

##### Health care costs

These include care by the general practitioner, allied health care, medical specialist, complementary and alternative medicine, hospitalisation, and medications. Data on resource use will be collected at a three monthly basis using retrospective questionnaires. Dutch standard costs will be used to value health care utilization [76]. If these are not available, prices according to professional organizations will be used. Medication use will be valued using unit prices provided by the Dutch Society of Pharmacy [77].

##### Productivity-related costs

Workplace productivity losses (i.e. work absenteeism and presenteeism) will be valued using salaries of the participants when using the employer's perspective and using average salaries per gender and five-year age group when using the societal perspective.

##### Participant costs

Since the intervention stimulates participants to engage in regular physical activity, self-reported costs related to sports activities (membership fees and sports equipment costs) will be collected on a three monthly basis.

#### Effect analysis

The effectiveness of the lifestyle intervention will be assessed using a regression analysis with the outcome measures at follow-up (6 months and 12 months) as the dependent variables and adjusting for the baseline levels of the outcome measure. Both crude and adjusted analyses will be performed. Linear and logistic (longitudinal) regression analyses will be performed using SPSS 18.0 (SPSS Inc. Chicago, Illinois, USA). According to the intention-to-treat principle, all available data of the participants will be used for data analysis. For all analyses, a two-tailed significance level of < 0.05 will be considered statistically significant.

#### Process evaluation

A process evaluation with the aid of the RE-AIM framework will be performed to evaluate the diverse intervention components [78]. The RE-AIM model assesses 5 dimensions: reach, efficacy, adoption, implementation, and maintenance. These dimensions interact to determine the impact of the programme. In addition, an adapted version of the framework of Steckler and Linnan will be applied [79]. The following process indicators will be measured in the first follow-up questionnaire (at 6 months after baseline) and continuously during the intervention period: context, recruitment, reach, dose delivered, dose received, satisfaction about the intervention, and fidelity.

#### Economic evaluation

The economic evaluation aims to determine the cost-effectiveness of the intervention compared with usual care from the societal and employer's perspective. Also, the cost-benefit will be determined from the employer's perspective. The time horizon will be one year, similar to the trial. Analyses will be performed according to the intention-to-treat principle. In the main analysis, missing data will be imputed using multiple imputation techniques [80]. Sensitivity analyses will be done to assess the robustness of the results.

First, the total societal and employer's costs will be estimated, and compared between the intervention and control group. The 95% confidence intervals will be estimated using approximate bootstrap confidence (ABC) intervals [81]. Societal costs include all cost measures described in the method section. From the employer's perspective, only costs relevant to the employer are included (i.e. intervention costs, other workplace health promotion costs, and productivity-related costs). For the cost-effectiveness analysis (CEA), incremental cost-effectiveness ratios will be calculated by dividing the difference in costs between both groups by the difference in effects on the primary outcome measures (societal perspective), and outcomes measures relevant to the company (employer's perspective). Bootstrapped cost-effect pairs will be graphically presented on cost-effectiveness planes [82]. Cost acceptability curves will be generated, showing the probability for cost-effectiveness of the intervention at different ceiling ratios. Also, a cost-benefit analysis (CBA) will be performed, in which the incremental intervention and other workplace health promotion costs will be compared to the incremental productivity-related costs.

## Discussion

The aim of this design article was to describe the development and plan for the evaluation of a (lifestyle) programme aimed at prevention and reduction of overweight and MSD among construction workers. This study may be of importance at company level to gain more insight in the effects of preventive measures, and to support decision making on which health promoting activities should be applied. Because the intervention is conducted in the occupational setting a large number of people can be reached, which may have an impact on health outcomes, and company as well as health care costs.

### Strengths

The intervention was designed following the IM protocol. This has been done before in health promotion interventions [83-85]. The development has been conducted with key figures in the organisation as well as with the target group aiming at a better compliance of employers and OHS with the VIP in construction protocol and allowing a scientific approach with consideration of daily practice. If the intervention proves to be effective, then the programme can be directly implemented.

Although the components of the intervention will not be evaluated separately, the process evaluation will give qualitative insight into the success factors, applicability and usefulness of the separate intervention components. Furthermore, the process evaluation outcomes can improve the programme before it will be really implemented.

### Limitations

Creating matrices in step 5 of the intervention mapping protocol was not fully applied, as this is a very time-consuming process. However, since the most important stakeholders were involved during the design of the study, it is expected that the adoption and implementation of the programme is ensured.

Health promotion efforts, particularly those directed to somewhat resistant workers who are at high risk, should preferably be integrated with the provision of improved working conditions. A systematic review of the effectiveness of health promotion interventions in the workplace concluded that participation in workplace health promotion may be increased if interventions also take into account health risks arising from work activities [86]. In this study, not all input of the intended target group has been implemented. This resulted from the fact that the programme has been developed in close cooperation with the management of the organisation, their approval was needed to carry out programme components. It is possible that the programme would have involved other components if only the input of the target group had been taken into account. However, this programme was developed with the intention to be implemented. Therefore, we believe that involving all important stakeholders is necessary.

Finally, this programme has been developed within a specific organisation. In this study, only stakeholders from the participating company and its OHSs were involved in the feasibility assessment and the focus group interviews. Also, a specific characteristic of the construction industry is that most employees are not working at a set location. The optimal infrastructure to reach workers is possibly different in other companies/branches. Therefore, it is possible that the IM process would have led to a different protocol in other workplace settings. This should be taken into account when implementing the intervention outside the construction industry. When generalising this programme to another context, the IM procedure can be applied to modify the existing programme.

## Conclusion

In conclusion, the development of the VIP in construction intervention resulted in a health programme tailored to the needs of construction workers. The method of IM provided the tools to do this systematically. If proven (cost-)effective the programme can be directly implemented, and with minor adaptations in other companies involving blue collar workers or companies that are already offering regular health screening. OHSs or human resource managers may incorporate this method in their usual prevention management. The results of the (process) evaluation will help policy makers decide which elements of the intervention can best be used.

The (cost-)effectiveness and the (implementation) process regarding this intervention will be evaluated. The results of this RCT will be available in 2012.

## Abbreviations

BMI: Body Mass Index; CBA: Cost-benefit Analysis; CEA: Cost-effectiveness Analysis; CVD: Cardiovascular Disease risk; HPQ: World Health Organisation -Health and Work Performance Questionnaire; IM: Intervention Mapping; MSD: Musculoskeletal Disorders; OHS: Occupational Health Services; OP: Occupational Physician; PA: Physical Activity; PAPM: Precaution Adoption Process Model; PHC: Personal Health Coach; PHS: Periodic Health Screening; RCT: Randomized Controlled Trial; SCORE: Systematic Coronary Risk Evaluation; SQUASH: Short Questionnaire to Assess Health Enhancing Physical Activity; TPB: Theory of Planned Behaviour; VIP: Vitality in Practice; WC: Waist Circumference.

## Competing interests

The authors declare that they have no competing interests.

## Authors' contributions

EV and KP were involved in developing the concept and the design of the study. LV and EV were involved in further developing the idea and the protocol for carrying out the study. LV was responsible for the data collection and she drafted the manuscript. All authors contributed to the final manuscript by reading and correcting draft versions. All authors read and approved the final manuscript.

## Pre-publication history

The pre-publication history for this paper can be accessed here:

http://www.biomedcentral.com/1471-2458/12/89/prepub
